# Lauric Acid Microemulsions Inhibit *Staphylococcus aureus* Through Cell Membrane Disruption and Potential Interference with Peptidoglycan Biosynthesis

**DOI:** 10.3390/foods15162867

**Published:** 2026-08-17

**Authors:** Peipei Ma, Runrun Zhang, Chen Li, Qiao He, Xinhui Zhang, Zhixiang Cai

**Affiliations:** 1 National-Local Joint Engineering Laboratory of Intelligent Food Technology and Equipment, Zhejiang Key Laboratory for Agro-Food Processing, Integrated Research Base of Southern Fruit and Vegetable Preservation Technology, College of Biosystems Engineering and Food Science, Zhejiang University, Hangzhou 310058, China; 12113042@zju.edu.cn (P.M.); 12113071@zju.edu.cn (R.Z.); 12513055@zju.edu.cn (C.L.); 2 Key Laboratory of Special Agri-Product Quality and Hazard Control Technology of Zhejiang Province, College of Life Sciences, China Jiliang University, Hangzhou 310018, China; heqiao@zju.edu.cn; 3 Zhejiang Collaborative Innovation Center for Full-Process Monitoring and Green Governance of Emerging Contaminants, College of Biological and Environmental Engineering, Zhejiang Shuren University, Hangzhou 310015, China; 4 New Sterilization Technology Joint Research Center, Future Food Laboratory, Innovation Center of Yangtze River Delta, Zhejiang University, Jiaxing 314100, China

**Keywords:** lauric acid, emulsion, *Staphylococcus aureus*, membrane-mediated antimicrobial mechanism, peptidoglycan, foodborne pathogen control

## Abstract

*Staphylococcus aureus* (*S. aureus*) is a prominent foodborne pathogen that poses a continuous threat to global public health and food safety due to its possession of a variety of toxins and its multidrug resistance. Medium-chain fatty acids (MCFAs), notably lauric acid (LA), exhibit strong antimicrobial properties, but their application is heavily constrained by poor water solubility. In this study, optimized LA emulsions stabilized by chitosan (CS) and polyvinyl alcohol (PVA) were evaluated for their antibacterial activity and detailed mode of action against *S. aureus* ATCC 6538. The antibacterial activities were evaluated by the maximum inhibition zone, with the 20 CS-PVA/DLTA-LA formulation exhibiting stable dispersion and potent antibacterial activity at 1%. The underlying antibacterial mechanisms against *S. aureus* were specifically focused on cell membranes and peptidoglycan. Therein, the binding of emulsion droplets to the anionic bacterial surface was driven by electrostatic attraction. Membrane degradation was also observed with membrane dysfunctions involving membrane depolarization, increased permeability, and fluidity reduction triggered by their subsequent insertion into the lipid bilayer, which may cause cell dysmetabolism, disintegration, and eventual cell death. Overall, these findings substantiate that LA emulsions disrupt *S. aureus* by operating potentially multi-targeted effects involving cell membrane disruption and peptidoglycan interference, offering a promising alternative approach warranting further investigation for foodborne pathogen control.

## 1. Introduction

Food safety and the mitigation of foodborne illnesses remain critical challenges that are faced by the global food industry and public health sectors. *Staphylococcus aureus* (*S. aureus*) is a ubiquitous Gram-positive foodborne pathogen that frequently causes extensive contamination and triggers severe food poisoning outbreaks through the ingestion of preformed, thermostable enterotoxins [1,2]. Traditionally, synthetic chemical preservatives have been widely deployed to inhibit microbial proliferation within food matrices. However, escalating consumer concerns regarding the potential long-term health risks of synthetic additives, coupled with increasingly stringent regulatory frameworks, have catalyzed an urgent transition toward natural, clean-label, and highly biocompatible antimicrobial strategies [3]. Among various natural bioactive candidates, medium-chain saturated fatty acids (MCFAs) and their derivatives have garnered profound interest due to their multi-target antimicrobial mechanisms, which primarily involve cell membrane disruption and metabolic dysfunction, positioning them as promising antimicrobial agents [4,5]. Specifically, lauric acid (LA), a natural 12-carbon fatty acid abundant in coconut oil and dairy products, exhibits remarkable bactericidal efficacy against Gram-positive pathogens such as *S. aureus*. This efficacy is predominantly driven by its capacity to compromise cell membrane integrity and alter membrane fluidity, which renders it an exceptional candidate for combating recalcitrant and recurrent *S. aureus* infections [6]. Additionally, LA has also been reported to destabilize multicellular aggregates, thereby inhibiting the formation of biofilm [7].

The notable antimicrobial efficacy of LA is counterbalanced by practical limitations in food applications, which arise from limited aqueous solubility, high hydrophobicity, propensity to crystallize at room temperature, and susceptibility to environmental degradation. Therefore, strategies such as chemical modification, complexation, and the fabrication of emulsion-based delivery systems have been proposed [8,9,10]. Nonetheless, many conventional preparation processes are limited by operational complexity, an inability to guarantee sustained bactericidal activity, and low loading capacities for LA. To address these drawbacks, lipid-based emulsion systems have been extensively explored [11,12]. These delivery architectures not only prevent fatty acid precipitation and facilitate long-term, stable dispersion in the aqueous phase, but also preserve the native biological activity of the encapsulated agent without chemical alteration. Furthermore, the fine droplets with a high specific surface area enhance the physicochemical interactions between the antimicrobial agent and microbial cells, thereby improving the overall functional performance.

Based on these findings, a series of formulations and processing parameters were systematically optimized in the present study, culminating in the development of a food-grade lauric acid-DLTA (a vitamin E derivative) emulsion. In this system, a chitosan–polyvinyl alcohol (CS-PVA) blend was utilized as the aqueous phase to provide interfacial stabilization and structural support for the emulsion network. The oil phase was composed of LA dissolved in vitamin E (VE) oil, which simultaneously augmented the antioxidant capacity; absolute ethanol was incorporated to enhance phase compatibility and dispersibility under ultrasound-assisted emulsification. The physicochemical properties, microstructure, and stability of the prepared emulsion were comprehensively characterized via dynamic light scattering (DLS), confocal laser scanning microscopy (CLSM), cryo-scanning electron microscopy (cryo-SEM), thermogravimetric analysis (TGA), and rheological measurements. The optimized emulsion exhibited excellent storage stability, with uniformly dispersed oil droplets forming a dense, cohesive network. Additionally, superior thermal stability and viscoelastic attributes were also demonstrated, and structural integrity was maintained throughout the storage period. Preliminary evaluations of its bioactivity revealed that a 1,1-diphenyl-2-picrylhydrazyl (DPPH) radical scavenging rate of 26.57% was achieved and a 2.8 log CFU/mL reduction in *S. aureus* was induced by the 1:10 (oil-to-water mass ratio) formulation at a 1% concentration within 2 h.

However, the precise antimicrobial mechanisms underlying this emulsion system remain to be fully elucidated. Recent insights have indicated that anti-staphylococcal fatty acids are likely to operate via complex, multi-target pathways, ranging from classical membrane disruption to emerging modes of gene regulation and signaling interference, rather than through simple, non-specific physical lysis [13]. This mechanistic diversity not only confers robust antimicrobial activity but also minimizes the likelihood of inducing bacterial resistance, thereby holding profound potential for biotechnological applications. The present study therefore sought to characterize the antimicrobial mechanisms of the optimized LA emulsions against *S*. *aureus* ATCC 6538, specifically exploring their potential multi-target interactions. The effects of the emulsion on cell surface charge, membrane potential, permeability, fluidity, nascent peptidoglycan biosynthesis, and bioenergetic status were investigated using systematic biophysical assays and advanced imaging techniques, including transmission electron microscopy (TEM), Fourier-transform infrared spectroscopy (FTIR), and real-time fluorescent D-amino acid (HADA) metabolic labeling. While this study demonstrates potent in vitro antimicrobial activity against *S. aureus*, the application of LA emulsions in actual food systems requires further validation under realistic food storage conditions.

## 2. Materials and Methods

### 2.1. Bacterial Strains and Culture Conditions

Single colonies of *S. aureus* ATCC 6538 on the Luria–Bertani medium (Hope Bio-Technology Co., Ltd., Qingdao, Shandong, China, HB0129) were inoculated into the Luria–Bertani broth medium (Hope Bio-Technology Co., Ltd., Qingdao, Shandong, China, HB0128) and incubated at 37 °C for 18 h with shaking (TS-2102C; Tensuc, Shanghai, China). The culture was subsequently centrifuged at 6000× *g* and 4 °C for 10 min to collect the bacterial cells and then washed twice with 0.85% sterile saline solution (Sodium chloride, Sinopharm Group Chemical Reagent Co., Ltd., Shanghai, China, cat. no. 10019318, AR grade solution) prior to experimental use.

### 2.2. Preparation of LA Emulsions

The preparation of LA emulsions was optimized as follows: LA (MW 200.32, Aladdin Reagent Co., Ltd., Shanghai, China, 143-07-7, 98%) was first blended with DLTA (Yuanye Biotechnology Co., Ltd., Shanghai, China, 52225-20-4, 99.60%) at a 1:2.5 mass ratio to generate a homogeneous oil phase. Subsequently, an equal quantity of absolute ethanol (Sinopharm Chemical Reagent Co., Ltd., Shanghai, China, 64-17-5, ≥99.9%) was added to the prior oil phase, and the mixture was stirred continuously for 30 min. The aqueous phase, containing 0.2 wt% CS (Shanghai Macklin Biochemical Co., Ltd., Shanghai, China, 9012-76-4, degree of deacetylation ≥ 95%) and 2 wt% PVA (Aladdin Reagent Co., Ltd., Shanghai, China, 9002-89-5, 98~99%), was subsequently added dropwise into the oil phase at aqueous-to-oil ratios of 1:20, 1:15, 1:10, and 1:5 (*v*/*v*), namely 20 CP/DL (CS-PVA/DLTA-LA), 15 CP/DL, 10 CP/DL, and 5 CP/DL, respectively, and the resulting mixtures were magnetically stirred for another 30 min. The coarse emulsions were further emulsified using a 20 kHz ultrasonic processor (Scientz-II D; Ningbo Scientz, Zhejiang, China) at 450 W in an ice bath for 15 min with a pulse cycle of 2 s on and 2 s off to yield the final emulsion. All experimental procedures were conducted under light-shielded conditions.

### 2.3. Zone of Inhibition (ZOI)

The antibacterial properties of the LA emulsions were evaluated through the Kirby–Bauer disk diffusion method. Overnight cultured *S. aureus* was diluted to 0.5 McFarland Turbidity Standard (McF) using sterile saline and was subsequently spread evenly onto Mueller–Hinton agar (MHA) (Solarbio, Beijing, China, cat. no. M8550) plates. Sterile Whatman No. 1 filter paper disks (6 mm in diameter) (Cytiva, Shanghai, China) were impregnated with 10 μL of each emulsion at concentrations of 1%, 3%, and 5% and were allowed to dry under aseptic conditions. Sterile saline was applied as a negative control. The prepared disks were then placed firmly onto the inoculated agar plates. Following incubation at 37 °C for 24 h, the antibacterial activity was determined by measuring the diameter of the clear zone of inhibition (ZOI) surrounding each disk. The diameter of each inhibition zone, inclusive of the 6 mm disk, was measured using a digital vernier caliper and recorded in millimeters (mm). All assays were performed in triplicate (*n* = 3), and the results were presented as mean ± standard deviation (SD).

### 2.4. Morphological Analysis of LA Emulsions-Treated Cells

For morphological characterization, *S. aureus* ATCC 6538 suspensions were subjected to 1% emulsion treatment for 0.5 h, followed by preparation for TEM observation. Bacterial cells were collected by centrifugation, fixed overnight in 2.5% glutaraldehyde at 4 °C, and dehydrated in graded ethanol (30%, 50%, 70%, 80%) and acetone (90%, 95%, 100%). Resin-embedded pellets were sectioned with an ultramicrotome (EM UC7, Leica, Vienna, Austria), stained with alkaline lead citrate and uranyl acetate, and imaged using TEM (H-7650, Hitachi, Tokyo, Japan).

### 2.5. ATP Activity

Changes in intracellular ATP levels in *S. aureus* after LA emulsions’ treatment were determined using an ATP assay kit (Beyotime Biotechnology, Shanghai, China, cat. no. S0026) [14]. Treated and untreated cells were collected by centrifugation, and then the cells were lysed by boiling in the lysis buffer for 2 min. The supernatant collected after centrifugation (12,000× *g*, 5 min, 4 °C) was used for further analyses, with sterile saline-treated bacteria as the control. ATP levels were quantified using a fluorescence microplate reader (Infinite^®^ E Plex, TECAN, Grödig, Austria) in luminance mode.

### 2.6. ROS Determination

LA emulsions (1%) were added to the bacterial suspension and incubated for 0.5 h. The treated and untreated bacteria were harvested by centrifugation and resuspended. A 20 µL aliquot of DCFH-DA (Solarbio, Beijing, China, 4091-99-0, ≥98%) was introduced into 2 mL of bacterial suspension, yielding a final concentration of 10 µM. Following a 20 min incubation at 37 °C, the cells were pelleted, washed twice, and resuspended in 0.85% sterile saline. Fluorescence intensity was then recorded on a microplate reader at excitation/emission wavelengths of 488/525 nm [15].

### 2.7. Analysis of Cell Membrane Integrity

To assess bacterial membrane integrity, the release of intracellular nucleic acids and proteins was monitored. Following treatment, bacterial suspensions (untreated and treated) were centrifuged at 8000× *g* for 10 min, and the resulting supernatants were collected. Nucleic acid leakage was quantified by absorbance at 260 nm, whereas protein leakage was determined by absorbance at 280 nm. The extent of leakage was expressed as OD_260_ and OD_280_ values, respectively [16].

### 2.8. Membrane Potential

To monitor alterations in bacterial membrane potential, the rhodamine 123 assay was employed. The dye (Aladdin Reagent Co., Ltd., Shanghai, China; 62669-70-9; ≥ 98%) was prepared in phosphate buffer and added to both untreated and treated cell suspensions at a final concentration of 2 μg/mL. After incubation at 37 °C for 30 min in the dark, the samples were centrifuged, washed twice, resuspended in sterile saline, and dispensed into black 96-well plates. The fluorescence signal was quantified using a plate reader, with the excitation and emission set at 480 nm and 530 nm, respectively [17].

### 2.9. Membrane Permeability

Membrane permeability damage was evaluated by measuring the uptake of propidium iodide (PI) (Sigma-Aldrich, Saint Louis, MO, USA, 25535-16-4, ≥94.0%). After treatment with 1% emulsions for 0.5 h, the bacterial pellet was collected using centrifugation at 4000× *g* for 10 min to remove dead cells and debris. The pellets were washed, re-suspended in sterile saline to an OD_600_ of 1.0, and then incubated with PI at a final concentration of 10 μM in the dark at 37 °C for 30 min. After two washes, the fluorescence (Excitation 535/Emission 617) was measured on a spectrophotometer [18].

### 2.10. Membrane Fluidity

Changes in membrane fluidity were evaluated by fluorescence anisotropy measurements. Treated *S. aureus* cells were pelleted by centrifugation and re-suspended in an equal volume of 2 µM 1,6-diphenyl-1,3,5-hexatriene (DPH) working solution (Sigma-Aldrich, Saint Louis, MO, USA; 1720-32-7; 98%). After 1 h dark incubation at 37 °C, the bacterial suspensions were transferred to a FlexStation 3 multimode microplate reader (Molecular Devices, LLC., San Jose, CA, USA) for anisotropy recording. Membrane fluidity was expressed as fluorescence polarization (*P*) using the following Equation (1):

(1) $$P=\frac{I_{VV}-GI_{VH}}{I_{VV}+GI_{VH}}$$

where *G* refers to the instrument grating factor, and *I_VV_*, and *I_VH_* denote the fluorescence intensities detected with vertical and horizontal analyzers, respectively, under vertically polarized excitation [19].

### 2.11. Conformational Properties of Bacterial Membrane Components

Fourier transform infrared (FTIR) spectroscopy (Nicolet 5700; Thermo Fisher Scientific, Waltham, MA, USA) was used to examine functional-group changes in bacterial membrane components. *S. aureus* pellets from emulsion-treated cultures were collected by centrifugation and subsequently lyophilized under vacuum. The dried samples were then analyzed via the KBr pellet method, with pure KBr (Aladdin Reagent Co., Ltd., Shanghai, China; 7758-02-3; ≥99%) serving as the background reference. The absorption spectra were acquired over 32 scans across 4000–400 cm^−1^ at a resolution of 4 cm^−1^ [20].

### 2.12. Confocal Laser Scanning Microscopy Imaging

Bacterial membrane integrity and peptidoglycan layer morphology were assessed by dual fluorescent staining. Treated cells were incubated with 20 μg/mL DAPI (Biofroxx, Einhausen, Germany, 28718-90-3, >98%) for 30 min and with 20 μg/mL Alexa Fluor 488-conjugated wheat germ agglutinin (WGA) (Thermo Fisher Scientific, Waltham, MA, USA, W11261) for 60 min at room temperature. The stained suspensions were then concentrated 10-fold, applied onto glass slides, and imaged using a confocal laser scanning microscope (CLSM) (ZEISS LSM 780, Carl Zeiss Microscopy GmbH, Jena, Germany) equipped with a 63× oil-immersion objective [21].

### 2.13. HADA Labeling

Peptidoglycan biosynthesis was monitored in real-time using the fluorescent D-amino acid 7-hydroxycoumarincarbonylamino-D-alanine probe HADA. An overnight culture of *S. aureus* was transferred to fresh nutrient broth containing 0.5% emulsions and incubated at 37 °C for 2 h to reach the logarithmic phase. The cells were then pelleted by centrifugation and resuspended to an optical density (OD_600_) of 0.5. HADA (Aladdin Reagent Co., Ltd., Shanghai, China, 2253733-10-5, ≥97%); these cells were then added to a final concentration of 250 μM, and this mixture was shaken at 37 °C for 30 min. After three washing steps, the labeled bacteria were resuspended in phosphate-buffered saline (pH 7.4) and imaged using a CLSM, with excitation at 350 nm and emission at 460 nm [22].

### 2.14. Statistical Analysis

Data are expressed as mean ± standard deviation (SD). One-way ANOVA was performed using SPSS 25.0 (SPSS Inc., Chicago, IL, USA), with statistical significance set at *p* < 0.05. Sterile saline as a non-bactericidal treatment control. Graphical representations were generated with GraphPad Prism 8.0.2 (GraphPad Software, San Diego, CA, USA) and Origin 2018 (OriginLab Corporation, Northampton, MA, USA).

## 3. Result and Discussion

### 3.1. Antibacterial Efficiency of LA Emulsions

The antibacterial activity of the LA emulsion was found to be positively correlated with concentration; however, an optimal concentration plateau was identified. Specifically, the maximum inhibition zone diameters were obtained at a total concentration of 3% across all treatment groups (10.0–11.2 mm), which were significantly larger than those observed at 1% (6.0–8.5 mm) and 5% (8.2–10.1 mm) (Table 1). This observation was consistent with the findings, which noted that the antimicrobial efficacy of glycerol monolaurate microemulsions positively correlated with concentration, with a peak zone of 16.1 mm against *S. aureus* at 12 mg/mL, after which the inhibition zone expansion decelerated [23]. This plateau effect is potentially driven by the self-aggregation of hydrophobic fatty acids or the structural densification of the emulsion. In addition, the disk diffusion method is influenced by agar diffusion capacity. The reduced zone at 5% may reflect impaired diffusion due to emulsion viscosity rather than diminished antimicrobial activity. Notably, 5 CP/DL achieved its maximum inhibition zones at both 3% and 5% concentrations. These findings are consistent with the report that eugenol-based microemulsions with larger droplet dimensions exhibited superior bactericidal activity against *S. aureus* compared to nanoemulsions [24]. Such enhanced performance was attributed to the sustained-release properties of the microemulsions, which facilitated prolonged bacterial contact, alongside a concentration-dependent potentiation of antimicrobial action.

However, at the 1% concentration, the inhibition zone of 5 CP/DL (6.0 mm) was statistically comparable to the control group, whereas 20 CP/DL, 15 CP/DL, and 10 CP/DL maintained potent antibacterial activities. As reported, the antibacterial performance of the mediated LA emulsion is governed not merely by its absolute payload, but critically by its encapsulation efficiency and release kinetics within the matrix [25]. At a lower total concentration (1%), the disproportionately high loading ratio in 5 CP/DL may induce heterogeneous distribution, localized precipitation, or compromised agar diffusion of LA emulsion. Conversely, an inhibition zone of 8.0 mm was sustained by 20 CP/DL at 1% concentration, demonstrating that an optimized loading ratio facilitates the stable dispersion of active ingredients within dilute emulsion systems.

This finding was corroborated by our previous study. At an emulsion concentration of 1%, all CP/DL formulations exhibited marked antimicrobial potency, with bactericidal performance concomitantly enhanced by increasing LA content and prolonged exposure. Upon raising the concentration to 3%, time-kill analysis revealed that each formulation achieved a rapid bacterial reduction of 2.5–3.0 log CFU/mL within 0.5 h, after which the curves reached a plateau.

### 3.2. Alteration of Bacterial Ultrastructure

**Figure 1:**
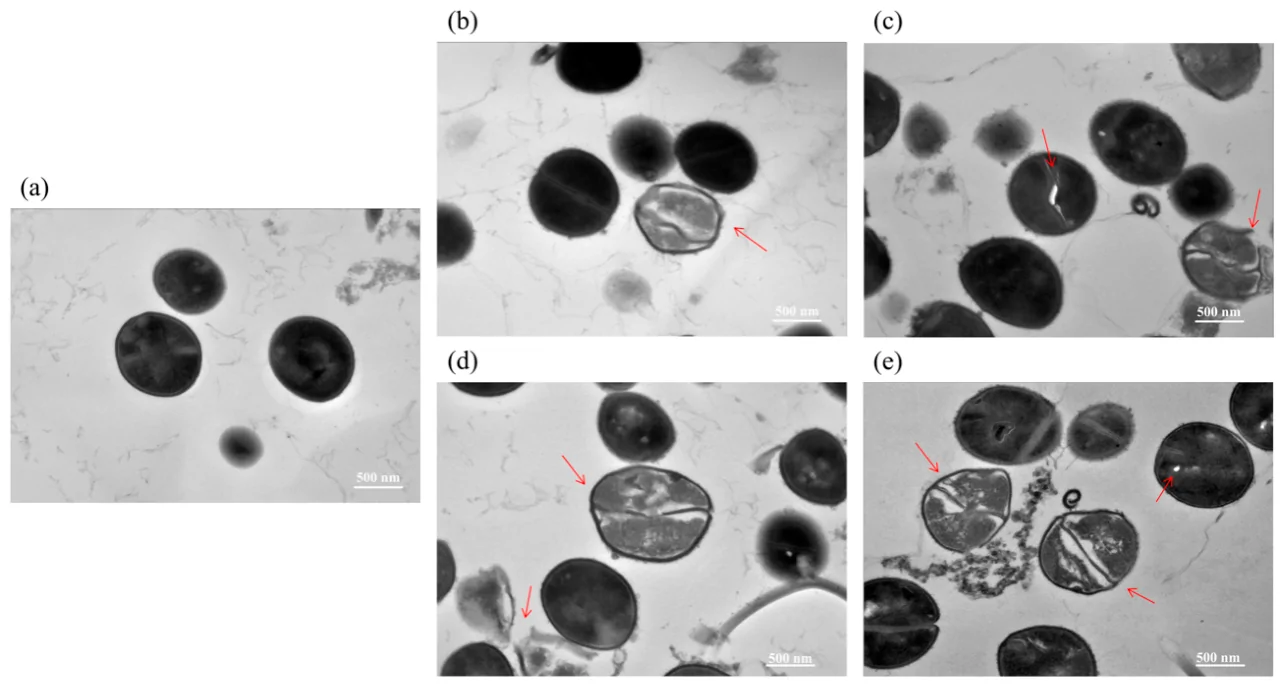
TEM image of *S. aureus* treated with different 1% emulsions for 0.5 h. Treatment-induced ultrastructural alterations in bacterial cells are denoted by arrows. Note: (**a**)—Control; (**b**)—20 CP/DL; (**c**)—15 CP/DL; (**d**)—10 CP/DL; (**e**)—5 CP/DL.

Ultrastructure analysis of bacteria following emulsion treatment was conducted to gain deeper insight into the effects of the emulsions on the bacterial cell envelope. As shown in Figure 1a, untreated cells were characterized by regular morphology, with uniformly dense cytoplasm and smooth, intact cell membranes. In contrast, following treatment with 1% emulsion for 0.5 h, marked ultrastructural alterations were observed in *S. aureus* cells, including irregular or disrupted cell membranes, unevenly distributed or vacuolated cytoplasm, distinct cytoplasmic banding, partially degraded cell walls, and cells with intact outer layers but internal damage. These changes are consistent with the ultrastructural modifications reported following treatment with other antimicrobial agents [26]. With increasing LA loading in the emulsion, progressively more severe cellular damage was observed in *S. aureus* (Figure 1b–e). These ultrastructural alterations suggested that LA molecules may insert into and disrupt the lipid bilayer of the bacterial cell membrane, thereby interfering with cellular metabolism and altering membrane functional properties.

### 3.3. Disruption of Bacterial Integrity and Membrane Functional Properties

**Figure 2:**
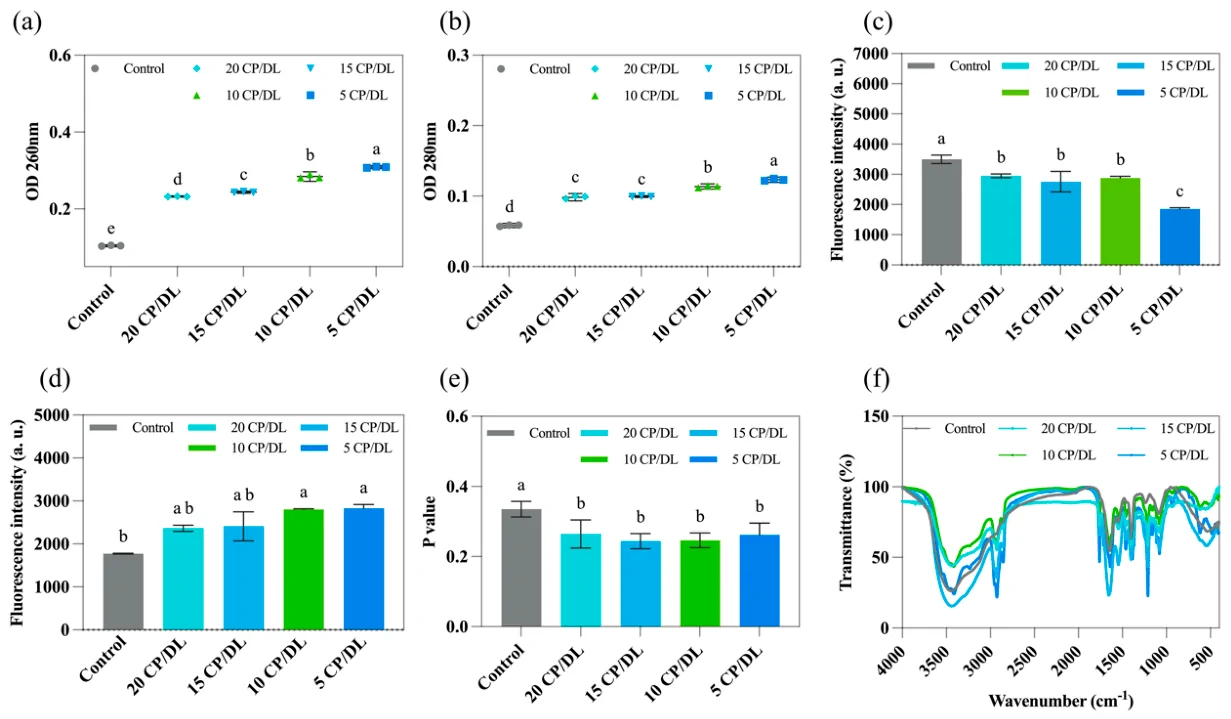
Nucleic acids (**a**) and protein (**b**) leakage from *S. aureus* 6538 after 0.5 h exposure to 1% emulsions. (**c**) Membrane potential of *S. aureus* 6538 measured by rhodamine 123 fluorescence after 0.5 h treatments with 1% emulsions. (**d**) Membrane permeability of *S. aureus* 6538 determined by PI uptake after treatments with 1% emulsions for 0.5 h. (**e**) Membrane fluidity of *S. aureus* 6538 evaluated by DPH fluorescence polarization after treatment with 1% emulsions for 0.5 h. (**f**) FTIR spectra of *S. aureus* 6538 treated with 1% emulsions for 0.5 h. All values are means ± SD (*n* = 3). Statistical significance was tested using one-way ANOVA; bars with different superscript letters indicate significant differences.

To further investigate the antimicrobial activity of LA emulsions against *S. aureus*, bacterial integrity and membrane functional properties were quantitatively assessed. Firstly, the leakage of intracellular contents (nucleic acids and proteins) before and after treatment was monitored by measuring the absorbance at 260 nm and 280 nm. The disruption of cell membrane integrity was performed to gain deeper insights into the bactericidal mode of action of the emulsion [27]. As illustrated in Figure 2a,b, a slight increase in the optical density was detected in the control group, which was likely attributed to natural cell death during the normal life cycle. Notably, compared with the control, a significant, concentration-dependent increase in the leakage of both nucleic acids and cellular proteins was observed after emulsion treatment, which indicated a progressive disruption of bacterial integrity with increasing LA content. The trend of intracellular content release was in good agreement with the results of the bactericidal efficiency and TEM observations, suggesting that the release of intracellular contents occurred synchronously with cell membrane disintegration. Hence, loss of cellular integrity is speculated to be one of the underlying mechanisms by which the emulsion inactivates bacteria.

To investigate the potential impact of emulsion treatment on membrane functionality, key functional properties, namely membrane potential, permeability, and fluidity, were assessed. These measurements were undertaken to further clarify the underlying antibacterial mechanism.

The impact of various emulsion treatments on bacterial membrane potential was evaluated using the lipophilic dye Rhodamine 123 (Rh123). Rh123 intracellularly accumulates in intact membranes and leaks upon membrane disruption; thus, its residual fluorescence directly reflects the bacterial membrane potential [28]. As shown in Figure 2c, treatment with LA emulsion suggested a significant reduction in the fluorescence intensity of *S. aureus* to approximately 15.7–47.0% of the control level (*p* < 0.05), signifying marked membrane depolarization. Because the *S. aureus* surface is inherently negatively charged, the emulsion droplets were likely adsorbed robustly onto the cell wall through electrostatic interactions, thereby interfering with the transmembrane potential [17]. This aligns with the findings that reported that exogenous fatty acids perturb the membrane potential of *S. aureus*, which represented a hallmark of membrane damage and dysfunction that governs bactericidal efficacy [29]. Overall, these observations corroborate the cell membrane disruption discussed above.

Consequently, membrane permeability was evaluated via propidium iodide (PI) uptake, a probe that selectively enters cells whose membranes are already compromised and fluoresces upon binding to nucleic acids [18]. As illustrated in Figure 2d, enhanced membrane permeability following emulsion treatment was clearly demonstrated by the increase in fluorescence intensity. As the primary bioactive ingredient, LA intercalates into the bacterial cytoplasmic membrane; at higher concentrations, membrane disruption is exacerbated, triggering increased permeability that culminates in cell lysis [6].

Optimal membrane fluidity, defined as the lateral mobility of lipids and proteins in the lipid bilayer, is essential for preserving cellular homeostasis. To gain deeper mechanistic insights into the emulsion’s bactericidal activity, membrane fluidity was monitored via DPH fluorescence polarization [30]. As shown in Figure 2e, the emulsion-treated cells exhibited a significant rise in fluorescence polarization (*p* < 0.05), which indicated a concomitant reduction in *S. aureus* bacterial membrane fluidity. Previous literature suggests that reduced membrane fluidity often reflects rigidification or altered packing of phospholipid acyl chains [31] and is intimately linked to membrane fatty acid profiles [29]. Consequently, the impact of the emulsion on membrane lipid composition warrants further investigation to thoroughly unravel its antimicrobial mode of action.

As FT-IR showed in Figure 2f, within the lipid-specific region, the intensity attenuation of the peaks at 2920 cm^−1^ and 2850 cm^−1^ (CH_2_ asymmetric and symmetric stretching, respectively) evidenced the interaction of LA emulsions with the phospholipid bilayer and consequent disruption of acyl chain ordering. As a 12-carbon saturated fatty acid, LA embeds into the membrane matrix, where it displaces or perturbs native phospholipid acyl chains, thereby weakening the lipid vibrational signals. It has been reported that spectral variations in this regime directly reflect lipid depletion or conformational disorder of fatty acid chains [32]. This was further corroborated by the weakened band at 1460 cm^−1^ (CH_2_ bending of membrane lipids), confirming diminished lipid content and a loss of molecular order. Simultaneously, in the membrane protein region, the decreased intensities at 1650 cm^−1^ (Amide I, C=O stretching) and 1540 cm^−1^ (Amide II, N–H bending) pointed to protein alterations [33]. Such suppression of amide bands following emulsion treatment typically signified protein denaturation or conformational shifts, which were triggered by compromised permeability and transmembrane potential dissipation. Moreover, pronounced spectral aberrations were observed in the phospholipid and cell wall skeletal regions. The peak near 1240 cm^−1^, associated with phosphodiester (PO_2_^−^) bonds in bilayers and nucleic acid phosphate backbones, was found to decrease in intensity, providing direct evidence of phospholipid structural degradation. A parallel trend was reported in *S. aureus* under carbon quantum dot stress, and it was ascribed to phospholipid damage [34]. For Gram-positive *S. aureus*, which possesses a cell wall heavily fortified with teichoic acid and peptidoglycan, spectral shifts in the C–O and PO_2_^−^ symmetric stretching at 1080–1050 cm^−1^ revealed that the emulsion simultaneously damaged the cell membrane and compromised the polysaccharide framework, resulting in cell wall loosening [35]. Remarkably, these spectral perturbations at 3300 cm^−1^ (hydrogen-bonding network), 2920 cm^−1^ (lipids), and 1240 cm^−1^ (phospholipids) were exceptionally pronounced in the 5 CP/DL group, highlighting extensive membrane and phospholipid damage at elevated LA payloads.

### 3.4. The Suppression of Energy Metabolism in S. aureus

**Figure 3:**
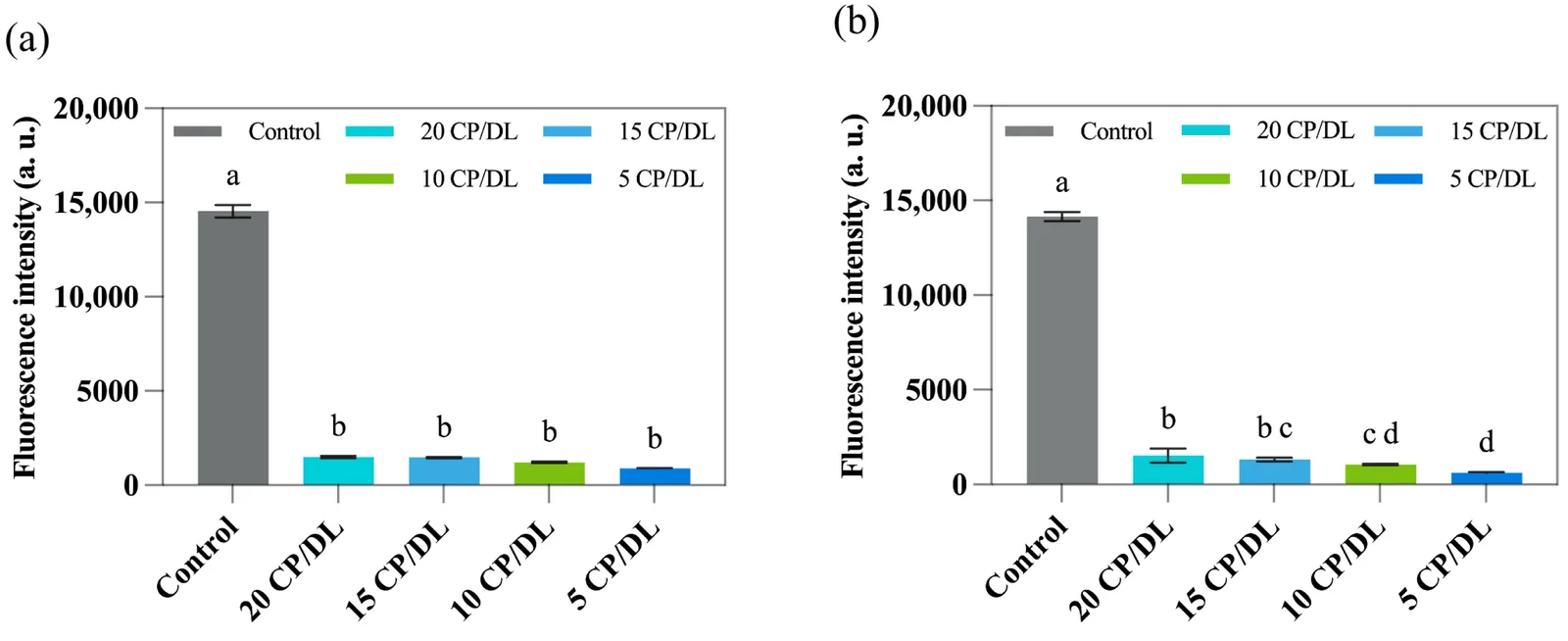
(**a**) ATP release and (**b**) ROS level of *S. aureus* after treatment with 1% emulsions for 0.5 h of different proportions. Statistical significance was tested using one-way ANOVA; bars with different superscript letters indicate significant differences.

LA emulsion treatment caused a precipitous decline in the intracellular ATP content of *S*. *aureus* (*p* < 0.001, Figure 3a), a phenomenon tightly coupled with ATP leakage driven by LA-mediated membrane disruption, as substantiated by our preceding results. This result corroborates the findings, in which it was demonstrated that membrane disruption by antimicrobial fatty acids (including LA) elicits the massive release of low-molecular-weight proteins and ATP-related contents in *S. aureus* [36]. Mechanistically, membrane depolarization and dissipation of the proton gradient (ΔpH) are triggered by LA intercalation. Given that ATP synthesis in *S. aureus* is driven by oxidative phosphorylation via the proton motive force (PMF), this depolarization deprives ATP synthase of its driving potential, thereby inhibiting ATP synthesis. Consistently, ATP leakage was identified as direct evidence of membrane damage in studies examining capric and LA-induced viability loss in persister cells [37]. However, the precipitous decline in intracellular ATP content may be attributed to membrane disruption and potential ATP leakage. Since extracellular ATP was not directly measured in this study, this interpretation remains speculative and requires further verification.

Intriguingly, intracellular ROS levels were markedly suppressed rather than elevated post-treatment (Figure 3b), suggesting that LA-mediated bactericidal efficacy may not be through the oxidative stress pathway, but rather through membrane disintegration and bioenergetic inhibition. Endogenous ROS predominantly stem from electron leakage within the electron transport chain (ETC); thus, when the ETC is structurally compromised by membrane disruption, ROS generation is substantially diminished [38]. The LA-induced membrane collapse abruptly halts electron transport, thereby quenching further ROS production. A finding supports the observations which confirmed that bacterial lethality becomes decoupled from ROS under conditions of carbon starvation or ETC suppression [39]. In addition, the reduction in ROS might reflect leakage due to cell membrane disruption, resulting in reduced measured values.

Distinct from antibiotics such as fluoroquinolones that trigger lethal ROS bursts, the amphiphilic molecules of LA directly insert into the phospholipid bilayer, leading to membrane depolarization, PMF dissipation, and ATP synthase malfunction. Consequently, the ETC is deactivated by structural impairment of the membrane, which reduces endogenous ROS generation and culminates in a synchronized drop in ATP and ROS. In agreement with the report that anthocyanin-laurate ester (C3G-LA) treatment caused a parallel 96% reduction in both membrane integrity and intracellular ATP in *S. aureus* [40], this model demonstrates consistent results. Collectively, these insights further validate the membrane-disruption and energy-exhaustion mode of action of LA-based agents.

### 3.5. The Interference with Peptidoglycan in S. aureus

**Figure 4:**
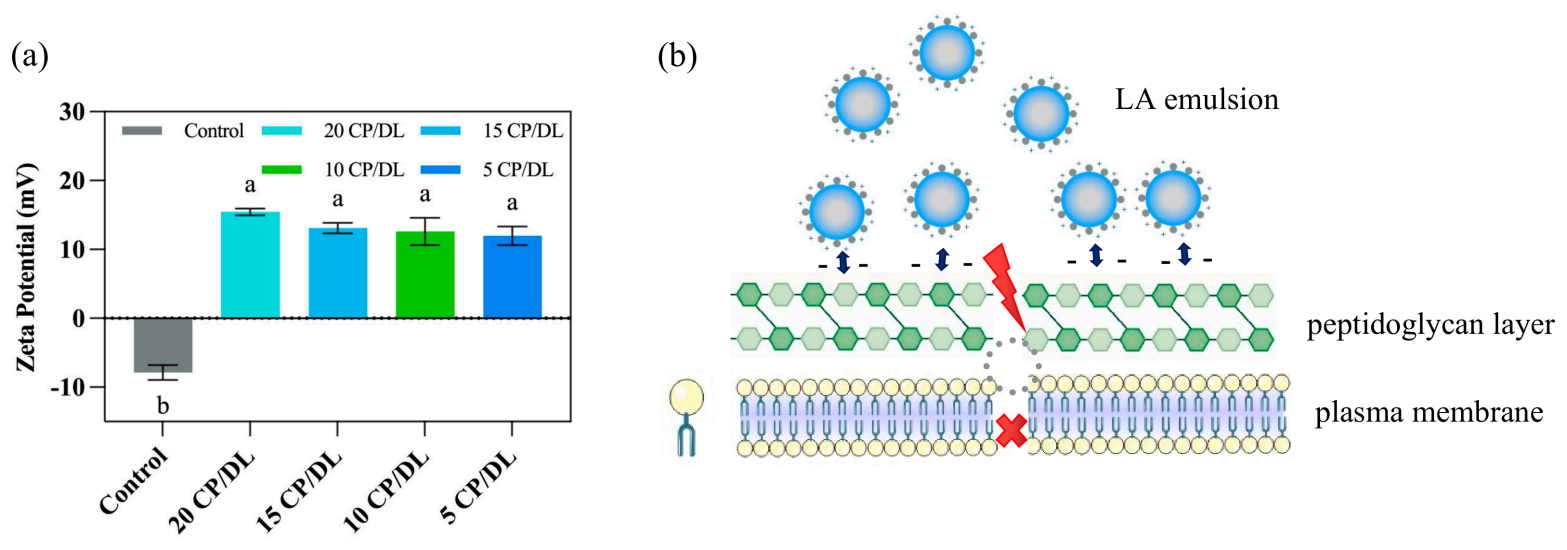
(**a**) Zeta potential of *S. aureus* 6538 bacterial suspension when exposed to different LA emulsions for 0.5 h. All values are means ± SD (*n* = 3). (**b**) Proposed interaction mechanism between *S. aureus* and LA emulsions.

Considering the distinct structural deformation and compromised membrane properties post-treatment, interfacial interactions between the bacterial surface and the emulsions were speculated to be responsible for the initial damage. The electrostatic component of these interactions was characterized by monitoring the zeta-potential shifts in *S. aureus* upon emulsion exposure [41]. The bacterial cell wall, which is inherently negatively charged, derives its electronegativity from teichoic acids with polyanionic phosphate backbones that are interwoven within the peptidoglycan matrix [42]. Therefore, the inherently cationic CP/DL emulsion droplets bound robustly to anionic *S. aureus* surface via electrostatic attraction. As a result of this absorption, the native bacterial charge was effectively shielded, and the net zeta-potential was reversed from −7.87 mV to positive values (Figure 4a). Since the concentration of chitosan is consistent across our different emulsion formulations, the charge reversal can be largely ascribed to the electrostatic association of the LA emulsion with the bacterial surface. As documented, zeta-potential perturbations directly reflected the magnitude of electrostatic affinity between lipid-based carriers and bacterial membranes [7]. It can be hypothesized that following initial electrostatic docking, the hydrophobic C12 alkyl chain of LA may insert into the peptidoglycan layer, potentially triggering downstream bactericidal cascades including membrane depolarization and permeabilization [43], which can be illustrated as Figure 4b proposed. Interestingly, no significant differences in surface charge were observed when the CP/DL ratio was varied, suggesting that the available binding sites on *S. aureus* had already been saturated by the cationic droplets. A charge-inversion plateau was thereby established, leading to a convergence of zeta-potential values above the threshold regardless of variations in antimicrobial efficacy [17].

**Figure 5:**
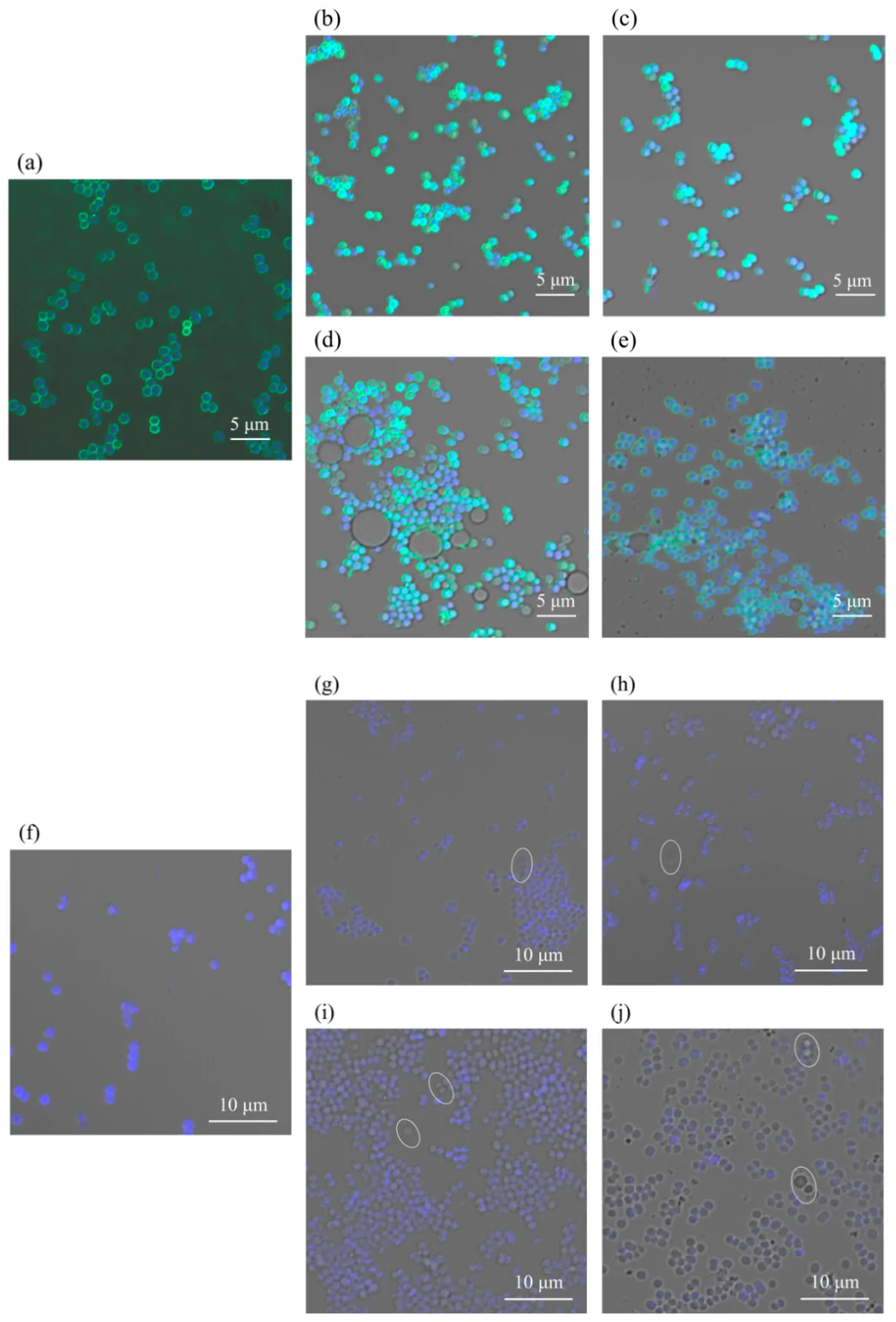
(**a**–**e**) CLSM images of *S. aureus* 6538 cells. DAPI and Alexa Fluor 488-conjugated wheat germ agglutinin were used to stain nucleic acids (blue fluorescence) and peptidoglycan (green fluorescence) of *S. aureus* cells with 1% emulsions for 0.5 h, respectively. (**f**–**j**) Visualization of PGN synthesis in *S. aureus* 6538. Emulsions-treated and untreated bacterial cells were incubated with fluorescent d-amino acid HADA and then visualized by CLSM. The regions of interest are marked with white circles. Note: (**a**,**f**)—Control; (**b**,**g**)—20 CP/DL; (**c**,**h**)—15 CP/DL; (**d**,**i**)—10 CP/DL; (**e**,**j**)—5 CP/DL.

This robust electrostatic interaction likely induces downstream impairment of membrane components. To elucidate these structural alterations, untreated and emulsion-treated cells were counterstained with DAPI and Alexa Fluor 488-labeled WGA for high-resolution confocal imaging. DAPI acts as a DNA-intercalating probe, while Alexa Fluor 488-WGA specifically targets *N*-acetylglucosamine residues in the peptidoglycan matrix [44]. As presented in Figure 5a, control *S. aureus* cells exhibited a homogenous green fluorescent ring outlining the intact peptidoglycan layer, which wrapped around a densely clustered blue fluorescent nucleoid. Conversely, emulsion exposure induced striking aberrations in WGA signaling, characterized by fluorescence attenuation and structural deformation, and was accompanied by diminished DAPI-stained nucleoid regions (Figure 5b–e). This observation directly reflected compromised peptidoglycan integrity and depleted nucleic acid content [22,45]. These observations suggest a hypothetical sequential mechanism in which peptidoglycan damage may precede or occur concurrently with membrane lysis; however, direct temporal evidence is required to confirm this sequence.

To determine whether peptidoglycan biosynthesis is perturbed by the emulsion, in situ labeling with 7-hydroxycoumarin-3-carboxylic acid 3-amino-D-alanine (HADA) was conducted. As a fluorescent D-amino acid probe, HADA actively incorporates into the termini of nascent PGN stem peptides [46]. The phenotypic responses of *S. aureus* exposed to the 0.5% emulsions were then visualized using CLSM (Figure 5f–j). As depicted in Figure 5g–j, a drastic reduction in fluorescence intensity was detected upon emulsion exposure, with a subset of cells remaining entirely unlabeled, evidencing a compromised peptidoglycan assembly pathway under 0.5% emulsion stress. We also found that LA emulsion treatment was associated with HADA fluorescence delocalizing from the septum to the peripheral cell wall and signal loss in *S. aureus*. The marked reduction and mislocalization of HADA fluorescence observed upon emulsion treatment phenocopies the pattern reported for established peptidoglycan synthesis inhibitors such as GP11 [47], suggesting that the emulsion may interfere with peptidoglycan biosynthesis. However, without a direct side-by-side positive control in our assay, we present this as a working hypothesis rather than definitive proof. Beyond the inhibition of peptidoglycan synthesis, this alteration in septal localization appears to be a critical mechanism underlying the inability to form a functional division septum and disrupting normal cytokinesis. Furthermore, aberrant morphological traits, including cell enlargement and cell wall skeletal collapse, were observed, corroborating our preceding findings. Consistent with established paradigms of peptidoglycan-targeted antimicrobials [48], these insights substantiate that the LA emulsions induce bacterial lethality by concurrently suppressing peptidoglycan metabolism and impairing membrane-associated biosynthetic machinery.

## 4. Conclusions

This study successfully demonstrated the potent antimicrobial capacity and delineated the multi-targeted mechanism of optimized LA emulsions against *S. aureus*. Concentration-dependent antibacterial action was observed, with maximal inhibition achieved at a 3% concentration, while the 20 CP/DL formulation showed superior structural stability and dispersion at low concentrations. Mechanistic investigations revealed a sequential, dual-targeted conduction. First, the cationic emulsion droplets securely bind to the negatively charged cell surface via electrostatic attractions, causing a net charge reversal. Simultaneously, the emulsion heavily targets the bacterial cell wall, compromising the polysaccharide framework and robustly inhibiting real-time peptidoglycan biosynthesis. This docking facilitates the intercalation of LA into the lipid bilayer, which directly triggers cell membrane depolarization, increased permeabilization, and reduced membrane fluidity, resulting in the massive leakage of intracellular nucleic acids and proteins. Subsequently, this membrane collapse impairs the electron transport chain and dissipates the proton motive force, leading to a sharp drop in intracellular ATP and suppression of ROS production. In summary, LA emulsions function as a highly efficient, multi-target antimicrobial agent that eradicates *S. aureus* through synchronous membrane disintegration and cell wall assembly inhibition, highlighting its great potential as an alternative intervention strategy for foodborne pathogen control.

## Figures and Tables

**Table 1:** Zone of inhibition (ZOI) of different LA emulsions (concentration of 1%, 3%, 5%) treatments against *S. aureus*.

Treatment Concentration	20 CP/DL (mm)	15 CP/DL (mm)	10 CP/DL (mm)	5 CP/DL (mm)
1%	8.00 ± 0.01 ^a^	8.50 ± 0.46 ^a^	8.53 ± 0.47 ^a^	ND
3%	10.03 ± 0.06 ^a^	10.67 ± 0.58 ^a,b^	10.90 ± 0.36 ^a,b^	11.23 ± 0.25 ^b^
5%	8.80 ± 0.53 ^a^	8.70 ± 0.46 ^a^	9.03 ± 0.15 ^a^	10.13 ± 0.23 ^b^

Note: All values are expressed as mean ± standard deviation (SD) based on three independent measurements (*n* = 3). One-way analysis of variance (ANOVA) was employed to compare the CP/DL groups at each concentration; significant differences (*p* < 0.05) are denoted by different superscript letters. The original diameter of the antibiotic disk is 6.00 mm. ND = Not Detected; indicates no antimicrobial diffusion.

## Data Availability

The original contributions presented in this study are included in the article. Further inquiries can be directed to the corresponding authors.
